# *Mycobacterium smegmatis* is a suitable cell factory for the production of steroidic synthons

**DOI:** 10.1111/1751-7915.12429

**Published:** 2016-11-02

**Authors:** Beatriz Galán, Iria Uhía, Esther García‐Fernández, Igor Martínez, Esther Bahíllo, Juan L. de la Fuente, José L. Barredo, Lorena Fernández‐Cabezón, José L. García

**Affiliations:** ^1^ Department of Environmental BiologyCentro de Investigaciones BiológicasConsejo Superior de Investigaciones CientíficasRamiro de Maeztu 928040MadridSpain; ^2^ MRC Centre for Molecular Bacteriology and InfectionDepartment of MedicineImperial College LondonLondonSW7 2AZUK; ^3^ Department of BiotechnologyGadea BiopharmaParque Tecnológico de LeónNicostrato Vela s/n24009LeónSpain

## Abstract

A number of pharmaceutical steroid synthons are currently produced through the microbial side‐chain cleavage of natural sterols as an alternative to multi‐step chemical synthesis. Industrially, these synthons have been usually produced through fermentative processes using environmental isolated microorganisms or their conventional mutants. *Mycobacterium smegmatis* mc^2^155 is a model organism for tuberculosis studies which uses cholesterol as the sole carbon and energy source for growth, as other mycobacterial strains. Nevertheless, this property has not been exploited for the industrial production of steroidic synthons. Taking advantage of our knowledge on the cholesterol degradation pathway of *M. smegmatis* mc^2^155 we have demonstrated that the *MSMEG_6039* (*kshB1*) and *MSMEG_5941* (*kstD1*) genes encoding a reductase component of the 3‐ketosteroid 9α‐hydroxylase (KshAB) and a ketosteroid Δ^1^‐dehydrogenase (KstD), respectively, are indispensable enzymes for the central metabolism of cholesterol. Therefore, we have constructed a *MSMEG_6039* (*kshB1*) gene deletion mutant of *M. smegmatis* MS6039 that transforms efficiently natural sterols (e.g. cholesterol and phytosterols) into 1,4‐androstadiene‐3,17‐dione. In addition, we have demonstrated that a double deletion mutant *M. smegmatis* MS6039‐5941 [*ΔMSMEG_6039* (*ΔkshB1*) and *ΔMSMEG_5941* (*ΔkstD1*)] transforms natural sterols into 4‐androstene‐3,17‐dione with high yields. These findings suggest that the catabolism of cholesterol in *M. smegmatis* mc^2^155 is easy to handle and equally efficient for sterol transformation than other industrial strains, paving the way for valuating this strain as a suitable industrial cell factory to develop *à la carte* metabolic engineering strategies for the industrial production of pharmaceutical steroids.

## Introduction

Androstenedione (4‐androstene‐3,17‐dione; AD) and androstadienedione (1,4‐androstadiene‐3,17‐dione; ADD) are key intermediates of microbial steroid metabolism (García *et al*., 2012). These compounds belong to the 17‐keto steroid family and are used as the starting materials for the preparation of different pharmaceutical steroids (Malaviya and Gomes, 2008; Donova and Egorova, 2012; García *et al*., 2012). These synthons can be produced by microbial side‐chain cleavage of cholesterol or phytosterols as an alternative to multi‐step chemical synthesis based on digoxigenin, a steroid found exclusively in the flowers and leaves of *Digitalis* plants, as a starting material. Industrially, AD and ADD have been produced through fermentative processes using wild microorganisms that have been subsequently modified and optimized by conventional mutagenic procedures (Donova *et al*., 2005; Andor *et al*., 2006; Donova and Egorova, 2012; García *et al*., 2012). *Mycobacterium* spp. NRRL 3805B and 3683B capable of forming AD and ADD from sterols, respectively, are examples of these mutants used at industrial scale (Donova *et al*., 2005; Donova and Egorova, 2012). A drawback of the AD and ADD industrial production based on these wild strains is the usual and concomitant accumulation of unwanted by‐products which hinder downstream processes. The use of molecularly defined mutants has been envisioned to avoid such drawbacks, but the lack of genetic data on the microbial catabolism of steroids has hampered the construction of genetically engineered mutants so far. Nevertheless, some attempts have been conducted to construct site‐directed mutant strains of *Rhodococcus* (i.e. the best characterized cholesterol‐degrading organism) to produce AD, ADD and 9α‐hydroxy‐4‐androstene‐3,17‐dione (9OH‐AD) from natural sterols (e.g. cholesterol or phytosterols), but these mutants have not been used at industrial scale yet (van der Geize *et al*., 2000, 2001a,b, 2002a,b, 2008; Wilbrink *et al*., 2011; Yeh *et al*., 2014).

**Figure 1:**
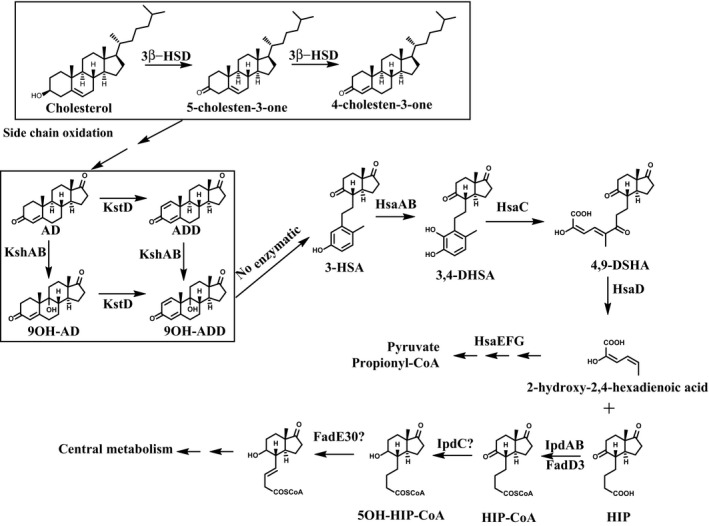
Proposed pathway for cholesterol degradation in *Mycobacterium smegmatis*. Cholest‐4‐en‐3‐one or any of the subsequent metabolites from degradation of the side‐chain up to (and including) AD may undergo a dehydrogenation reaction to introduce a double bond in the position 1, leading to compound cholest‐1,4‐diene‐3‐one in the case of cholest‐4‐en‐3‐one, or to the corresponding 1,2‐dehydro derivatives for other molecules. The side‐chain degradation of this compounds will be identical to that of the cholest‐4‐en‐3‐one to the common intermediate 9α‐hydroxyandrosta‐1,4‐diene‐3,17‐dione (9OHAD). 3β‐hydroxysteroid dehydrogenase (3β‐HSD), 3‐ketosteroid‐Δ^1^‐dehydrogenase (KstD), 3‐ketosteroid‐9α‐hydroxylase (KshAB), 3‐hydroxy‐9,10‐secoandrosta‐1,3,5(10)‐trien‐9,17‐dione (3‐HSA), 3,4‐dihydroxy‐9,10‐secoandrosta‐1,3,5(10)‐trien‐9,17‐dione (3,4‐HSA), 4,5,9,10‐diseco‐3‐hydroxy‐5,9,7‐trioxoandrosta‐1(10),2‐diene‐4‐oic acid (4,9‐DSHA), 3aα‐H‐4α(3′‐propionic acid)‐7aβ‐methylhexahydro‐1,5‐indanedione (HIP), 3aα‐H‐4α(3′‐propionic acid)‐5α‐hydroxy‐7β‐methylhexahydro‐1‐indanone (5OH‐HIP), 3‐hydroxy‐9,10‐secoandrosten‐1,3,5(10)‐trien‐9,17‐dione dyoxigenase (HsaC), 4,5,9,10‐diseco‐3‐hydroxy‐5,9,7‐trioxoandrosta‐1(10),2‐diene‐4‐oic acid hydroxylase (HsaD), 2‐hydroxy‐2,4‐hexadienoic acid hydratase (HsaE), 4‐hydroxy‐2‐hydroxy‐2‐ketovalerate aldolase (HsaF), acetaldehyde dehydrogenase (HsaG), HIP‐CoA transferase (LpdAB and FadD3), FadE30 (Acyl‐CoA dehydrogenase).

To create *à la carte* mutants able to produce AD and ADD from cholesterol or phytosterols we have tested *Mycobacterium smegmatis* mc^2^155 as a model strain based on our current knowledge on sterol catabolism in this microorganism (Fig. 1) (García *et al*., 2012). The 3‐ketosteroid 9α‐hydroxylase (Ksh) has been proposed as the key enzyme for ring‐B opening, and therefore, the removal of this activity should render ADD as end‐product. The Ksh enzymes of *Rhodococcus* and *Mycobacterium* have been characterized as two‐component monooxygenases, composed of an oxygenase (KshA) and a ferredoxin reductase (KshB) (van der Geize *et al*., 2002a; Capyk *et al*., 2009, 2011; Petrusma *et al*., 2009, 2012, 2014; Hu *et al*., 2010; Bragin *et al*., 2013; Penfield *et al*., 2014). Therefore, theoretically, a deletion mutant of one of the Ksh encoding genes, i.e. *kshA* or *kshB*, should accumulate ADD. On the other hand, 3‐ketosteroid Δ^1^‐dehydrogenase (KstD) is the key enzyme that transforms AD into ADD and thus, strains lacking KstD should theoretically accumulate 9OH‐AD. Finally, double mutants in Ksh and KstD should render AD as the main product for sterol degradation (Fig. 1) (García *et al*., 2012).

Nevertheless, these previous assumptions cannot be always easily confirmed by gene inactivation since cholesterol catabolic pathway usually presents functional redundancy for some steps of the pathway; this means that one catalytic step can be carried out by several homologous enzymes. Two KstD enzymes were reported in *Rhodococcus erythropolis* SQ1 (van der Geize *et al*., 2000) and accordingly, targeted disruption of only one of the *kstD* genes did not result on the accumulation of intermediates, but the strain lacking both dehydrogenase activities was able to convert AD into 9OH‐AD stoichiometrically (van der Geize *et al*., 2002b). Similarly, Fernández de las Heras *et al*. (2012) have demonstrated the existence of three KstD activities in *Rhodococcus ruber* strain Chol‐4 and showed that the triple KstD mutant was able to convert AD into 9OH‐AD. However, these mutants were unable to accumulate 9OH‐AD from cholesterol or phytosterols (van der Geize *et al*., 2001a; Fernández de las Heras *et al*., 2012). Brzostek *et al*. (2009) have identified six putative KstD enzymes within the *M. smegmatis* genome and a targeted disruption of one of them (KstD1) resulted in partial inactivation of the cholesterol degradation pathway and the consequent accumulation of AD. More recently, Wei *et al*. (2010) have identified and deleted a *kstD* gene (named *ksdD*_*M*_) in *Mycobacterium neoaurum* NwIB‐01, a strain isolated from a sterol contaminated soil that naturally accumulates ADD from phytosterols. Interestingly, the resulting deleted mutant (named NwIB‐2) was able to accumulate AD from phytosterols, but ADD is still present in the culture medium, suggesting that this strain contains other KstD enzymes (Wei *et al*., 2010). Three homologues of KstD have been characterized in *M. neoaurum* ATCC 25795 and thus, single deletion of these genes failed to result in a stable and maximum accumulation of 9OH‐AD due to residual KstD activities (Yao *et al*., 2014).

On the other hand, van der Geize *et al*. (2002a, 2008) have demonstrated that Ksh mutants of *R. erythropolis* SQ1 deleted in *kshA* or *kshB* genes can produce ADD from AD, but surprisingly they do not accumulate AD, ADD or other metabolites during sterol conversion. Interestingly, the degradation of phytosterols was not impaired in a *kshA* ^*‐*^ mutant and rates of degradation were comparable to those of the parent strain, suggesting that *Rhodococcus* has alternative enzymes to catabolize phytosterols. Moreover, the *kshB* ^*‐*^ mutant failed to cleave the side‐chain of sterols and, although phytosterols were oxidized to their stenone derivatives, they were not metabolized further, suggesting that *kshB* could be also involved in the degradation of sterol side‐chain in *Rhodococcus* (van der Geize *et al*., 2002a). The situation can be more complex taking into account that some strains of *Rhodococcus* may contain up to five KshA homologous proteins, each displaying unique steroid induction patterns and substrate ranges, confirmed that the 9α‐hydroxylation can take place at different steps of steroid oxidation (Petrusma *et al*., 2011).

On the basis of our knowledge on cholesterol metabolism in *M. smegmatis* (Uhía *et al*., 2012) we detected that this organism has several important differences with the sterol catabolism in *Rhodococcus* and we anticipated that these differences might render *M. smegmatis* as a suitable cell factory for the metabolic manipulation of this pathway. The results presented below demonstrate this hypothesis and show that *M. smegmatis* is an ideal cell factory to develop metabolic engineering strategies for the industrial production of AD, ADD or other steroid intermediates using natural sterols as feedstock.

## Results

### Identification of *Ksh* and *KstD* enzymes in *M. smegmatis*

In contrast to the frequent observation that the components of bacterial multicomponent oxygenases are encoded in a single operon, the *kshA* and *kshB* genes encoding Ksh activity in *Rhodococcus* (van der Geize *et al*., 2002a) or in *M. tuberculosis* (Cole *et al*., 1998) are located far from each other in the genome. Nevertheless, using the sequence of the annotated *kshA* and *kshB* genes from *Rhodococcus* (van der Geize *et al*., 2002a, 2008) and *M. tuberculosis* (Capyk *et al*., 2009) as probes, we have localized the corresponding orthologues in *M. smegmatis* (Table 1). This analysis revealed that there are at least two genes encoding putative oxygenase components, named *kshA1* and *kshA2*, and two genes encoding putative reductase components, named *kshB1* and *kshB2*. This means that *M. smegmatis* could produce theoretically four different Ksh hydroxylases, i.e. Ksh1 (KshA1B1), Ksh2 (KshA2B2), Ksh3 (KshA1B2), Ksh4 (KshA2B1). However, a detailed analysis of the upstream sequences of these genes showed that only the *MSMEG_5925* (*kshA1*) and *MSMEG_6039* (*kshB1*) genes are preceded by promoters inducible by cholesterol, having the consensus operator sequence for the binding of the KstR repressor, one of the regulators of cholesterol catabolism (named KstR regulon) (Kendall *et al*., 2007). Moreover, microarray expression experiments carried out on *M. smegmatis* indicate that *kshA1* and *kshB1* are induced 1.9‐fold and 7.0‐fold, respectively, in cholesterol with respect to glycerol (Table 1) (Uhía *et al*., 2012). On the contrary, *kshA2* and *kshB2* are not induced by cholesterol under the tested conditions (Table 1) (Uhía *et al*., 2012). These results strongly suggest that most probably only Ksh1 encoded by *kshA1B1* is involved in the metabolism of cholesterol in *M. smegmatis*.

**Table 1:** *In silico* analysis of *M. smegmatis* mc^2^ 155 genome

Protein	Gene^a^ *M. smegmatis* mc^2^ 155	Protein (aa length)	Identity^b^ (%)	KstR‐regulon operator	Induction fold^c^
KshA	*MSMEG_5925*	383	67	Yes	7.00
	*MSMEG_2870*	386	53	No	1.00
KshB	*MSMEG_6039*	353	63	Yes	1.88
	*MSMEG_2893*	351	56	No	1.21
KstD	*MSMEG_5941*	566	65	Yes	13.00
	*MSMEG_4864*	587	39	No	‐1.46
	*MSMEG_2869*	558	40	No	‐1.17
	*MSMEG_2867*	522	31	No	‐1.14
	*MSMEG_4870*	546	30	No	‐1.35
	*MSMEG_5835*	547	17	No	1.14

a Gene identifications correspond to the last annotation of *M. smegmatis* genome and are different to the annotations described by Brzostek *et al*. (2005).

b Identities were established using the proteins of *R. jostii* RHA1 as reference.

c Induction fold was calculated comparing gene expression in *M. smegmatis* cultured in cholesterol verus glycerol containing media (Uhía *et al*., 2012).

Among the six putative *kstD* genes identified in the *M. smegmatis* genome by Brzostek *et al*. (2009) that might encode the KstD‐like activity only the *MSMEG_5941* (*kstD1*) gene is controlled by a promoter containing the consensus KstR operator sequence and is differentially expressed in the presence of cholesterol (Table 1) (Uhía *et al*., 2012). This observation suggests that in *M. smegmatis* most probably only *kstD1* was involved in the catabolism of cholesterol.

Therefore, based on these analyses we anticipated the hypothesis that in *M. smegmatis* a single deletion of *kshB1* (or *kshA1*) and a double deletion of *kshB1* (or *kshA1*) and *kstD1* might be sufficient to generate mutants able to accumulate ADD or AD, respectively, when cultured in the presence of sterols.

### Construction of the *ΔkshB1 M. smegmatis* mutant

As mentioned above, to eliminate the two‐component Ksh1 hydroxylase activity (KshA1B1) in *M. smegmatis*, we assumed that it was enough to delete the *MSMEG_6039* (*kshB1*) gene encoding the reductase component, thus we constructed the MS6039 mutant (*ΔkshB1*) (Fig. S1). Ksh1 activity can be eliminated either by deleting *kshB1* or *kshA1* (or both), but we decided to test the single deletion of *kshB1* instead of *kshA1*, because in the absence of the corresponding oxygenase component, the reductase component of multicomponent oxygenases usually acts as a gratuitous scavenger of reduction power, generating futile cycles that might reduce the efficiency of the biotransformation process (Galán *et al*., 2000; Blank *et al*., 2010) and therefore, in this sense, it can be more convenient to suppress the reductase instead the oxygenase component. However, it is also true that other reductase enzymes present in the cell could replace their function. Nevertheless, we also decided to delete *kshB1* because the effect of the deletion of the *kshB* homologous gene has already been analysed in *Rhodococcus* (van der Geize *et al*., 2002a,b) and we would like to compare the performance of *M. smegmatis* having the same gene deletion.

**Figure 2:**
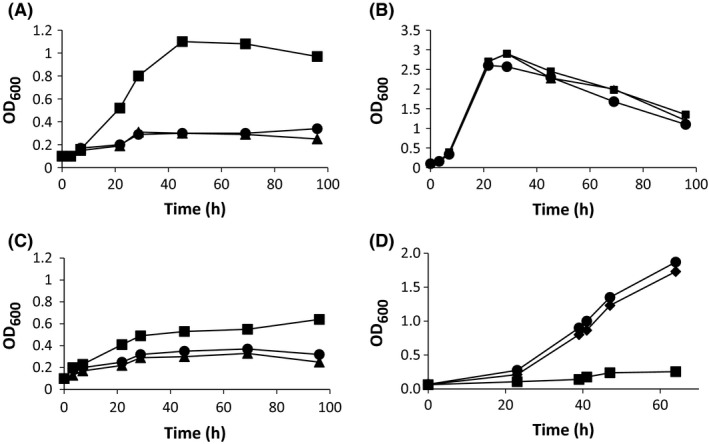
Growth curves of *M. smegmatis* mc^2^155, mutants and complemented strains cultured in shake flasks with different carbon sources. (A) Strains cultured with 0.4 g l^−1^ of cholesterol (mc^2^155 (squares), MS6039 (circles) and MS6039‐5941 (triangles)); (B) Strains cultured with 18 mM glycerol (mc^2^155 (squares), MS6039 (circles) and MS6039‐5941 (triangles)); (C) Strains cultured with 0.4 g l^−1^ of phytosterols (mc^2^155 (squares), MS6039 (circles) and MS6039‐5941 (triangles)); (D) Gene complementation of MS6039 mutant strain cultured with 1.8 mM of cholesterol (mc^2^155 (pMV261) (diamonds), MS6039 (pMV261) (squares) and MS6039 (pMV6039) (circles)). The data reported are the averages of three different assays.

The engineered MS6039 mutant (*ΔkshB1*) was unable to efficiently grow in cholesterol or phytosterols as a sole carbon and energy source when compared with the wild‐type strain (Fig. 2A). However, the mutant perfectly grows using glycerol as a carbon source (Fig. 2B). To confirm that the deletion of *kshB1* was responsible for the observed phenotype, we transformed the MS6039 mutant with the plasmid pMV6039 harbouring the *kshB1* gene. Figure 2D shows that the complemented MS6039 (pMV6039) strain recovered the capacity to grow in sterols as a sole carbon and energy source. These results confirmed that *kshB1* plays an essential role in the catabolism of sterols in *M. smegmatis*. In addition, considering that the MS6039 mutant is unable to efficiently grow in sterols even at long incubation periods, we can conclude that the KshB1 reductase activity of Ksh1 cannot be replaced by other mycobacterial reductases, i.e. KshB2 or other KshB‐like reductases, in the tested conditions, either because they are not expressed in these conditions or because they cannot interact with the KshA1 oxygenase component.

These experiments also revealed that wild‐type *M. smegmatis* is able to use efficiently different phytosterols as a carbon and energy source (Fig. 2C), and this is remarkable because the capacity of this strain to grow on a mixture of phytosterols has not been well documented in the literature so far.

On the other hand, the slight growth of MS6039 in the presence of sterols suggested that the mutant was able to degrade their side‐chain and predicted a possible accumulation of metabolic intermediates in the culture medium. As expected, Figure 4 shows the accumulation of ADD in the culture medium of MS6039 mutant when cultured in shake flasks, whereas *M. smegmatis* wild‐type strain completely mineralizes cholesterol (or phytosterols) and does not accumulate any intermediate in these culture conditions (data not shown). The ADD molar yield for the transformation of cholesterol or phytosterols was 91% for both feedstocks. Nevertheless, we have also detected the presence of small amounts of 22‐hydroxy‐23,24‐bisnorchol‐1,4‐dien‐3‐one (1,4‐HBC, 20‐hydroxymethylpregna‐1,4‐dien‐3‐one) as a by‐product (Fig. 4C and D).

In addition, we have tested the production of ADD from phytosterols in 5‐ and 20‐l stirred jar bioreactors using higher concentrations of phystosterols (20 g l^−1^) as substrate to study the industrial potential of MS6039 mutant strain. By carrying several replicates of these experiments in different operational conditions, we concluded that between 55% and 70% of added phytosterols are consumed and the molar yield to ADD varies from 67% to 80%, depending on the bioreactor scale. AD was also obtained in a yield from consumed phytosterols between 22% and 30%; other by‐products, such as 1,4‐HBC were detected as traces (Fig. S2). These experiments carried out at high sterol concentrations showed a significant contamination of unconverted AD suggesting that KstD activity is a bottleneck for the complete transformation of phytosterols into ADD at industrial scale. This problem can be overcome by changing the operational conditions (e.g. preinoculation growth, inoculation conditions, bioreactor agitation configuration, aeration flow, composition of culture medium, pH control, substrate additions, etc.) in combination with the overexpression of *kstD* genes in MS6039 that lead to a significant increase in ADD/AD ratio (Gadea personal communication).

The results contrast with the data obtained with the equivalent *kshB* ^*‐*^ mutant of *R. erythropolis* strain SQ1 (RG4 mutant), which only accumulates sitostenone from β‐sitosterol, because apparently, the RG4 mutant does not degrade the side‐chain of β‐sitosterol. To explain this performance, it was proposed that the KshB reductase from *R. erythropolis* was not only involved in the production of 9OH‐AD, but also in sterol side‐chain removal (van der Geize *et al*., 2002b). In *M. smegmatis*, the absence of KshB1 does not hinder the complete degradation of the sterol side‐chain (see Discussion).

### Construction of the *ΔkshB1* and *ΔkstD1 M. smegmatis* double mutant

According to our previous genomic analysis, it should be possible to produce AD from sterols in *M. smegmatis* by eliminating the KstD activity in the MS6039 mutant. Therefore, to test this hypothesis, we constructed the *M. smegmatis* MS6039‐5941 double mutant (*ΔkstD1*,* ΔkshB1*) by deleting the *MSMEG_5941* (*kstD1*) gene in the MS6039 (*ΔkshB1*) mutant (Fig. S1). As expected, the MS6039‐5941 (*ΔkshB1‐ΔkstD1*) double mutant was unable to efficiently grow in cholesterol or phytosterols as the sole carbon and energy source, but the double mutant grows in glycerol at the same rate than the wild‐type strain (Fig. 2B).

Accordingly to our predictions, when the MS6039‐5941 double mutant was cultured in minimal media containing glycerol as carbon and energy source and cholesterol (or phytosterols) as feedstock, the culture yields a large accumulation of AD in shake flasks (Fig. 4A and B). The AD molar yields for sterol transformations were 90% and 84% when cholesterol or phytosterols were used as substrates respectively. Interestingly, in these culture conditions, small amounts of 22‐hydroxy‐23,24‐bisnorchol‐4‐en‐3‐one (4‐HBC, 20‐hydroxymethylpregna‐4‐en‐3‐one) were accumulated during the biotransformation (Fig. 4C and D).

These results support the hypothesis that although *M. smegmatis* has six putative KstD dehydrogenases, the KstD1 enzyme encoded by the *MSMEG_5941* gene is the main KstD used for sterol catabolism in this bacterium. This observation contrasts with other cholesterol‐degrading bacteria, where several KstD dehydrogenases are involved in the metabolism of sterols (van der Geize *et al*., 2000, 2002b; Fernández de las Heras *et al*., 2012; Yao *et al*., 2014).

The MS6039‐5941 double mutant was tested in 2‐l jar bioreactor in the presence of 10 g l^−1^ of phytosterols. An almost complete transformation of phytosterols into AD (88–90%), 4‐HBC (10–11%) and very small amounts of ADD and 1,4‐HBC were detected in several replicates of this experiment (Fig. S3).

Interestingly, the accumulation of small amounts of ADD observed in the bioreactor experiments suggests that one of the other identified *kstD2*,* kstD3*,* kstD4*,* kstD5 or kstD6* homologous genes (Table 1) is somewhat active in the mutant. Thus, to further improve the AD production yield, this residual KstD activity should be identified and eliminated. Moreover, in this sense, it will be also important to understand why the three last carbons at C‐17 are not efficiently converted into propionyl‐CoA, rendering 4‐HBC and 1,4 HBC as by‐products of the pathway.

## Discussion

The first remarkable finding presented above is the experimental demonstration that *M. smegmatis* mc^2^155 is able to efficiently metabolize mixtures of phytosterols as carbon and energy sources through the same catabolic pathway utilized to mineralize cholesterol (Fig. 1) (Uhía *et al*., 2012). This finding is important because it supports the proposal of considering *M. smegmatis* as a useful cell factory for the industrial production of steroidic synthons from raw sterols. Surprisingly, in spite of the large accumulated knowledge on *M. smegmatis* mc^2^155 biology, this organism has not been used as a cell factory for this industrial purpose so far. The only case reported in the literature is an antibiotic resistant mutant deposited as *M. smegmatis* VKPM Ac‐1552 in the Russian National Collection of Industrial Microorganisms (VKPM) that appears to transform sterols into AD (Russian Federation Patent no. 2 126 837 (1999), reviewed in Donova (2007)).

The observation that the growth of *M. smegmatis* mc^2^155 in sterols is impaired by a deletion of the *kshB1* gene (*MSMEG_6039*) suggests that KshB1 is the main reductase component of the two‐component KshAB 9α‐hydroxylase in the cholesterol degradative pathway of this organism. Apparently, this reductase activity is not redundant in *M. smegmatis* and thus, it cannot be replaced by similar enzymes, as it has been demonstrated for other key enzymes of the pathway (Uhía *et al*., 2011). In addition, the *kshB1* gene does not appear to be critical for the degradation of the side‐chain of sterols in *M. smegmatis*, as suggested in *Rhodococcus* (van der Geize *et al*., 2002a), and therefore, its absence does not impair an efficient transformation of sterols into AD or ADD in the mutant strain.

On the other hand, the significant accumulation of AD in the MS6039‐5941 (*ΔkshB1‐ΔkstD1*) double mutant ascribes a fundamental role to the *kstD1* gene (*MSMEG_5941*) in the catabolism of cholesterol. Nevertheless, in this mutant, we have observed some enzyme redundancy because we were able to detected small amounts of ADD when high concentrations of phytosterols are transformed. Therefore, we can conclude that at least one of the other five putative KstD enzymes identified in *M. smegmatis* mc^2^155 (Table 1) can also replace this function, but this alternative KstD activity is very low and does not appear to fulfil the relevant function observed in other cholesterol‐degrading strains (van der Geize *et al*., 2000, 2002b; Fernández de las Heras *et al*., 2012; Yao *et al*., 2014).

The results presented in this work suggest that the metabolism of sterols in *M. smegmatis* mc^2^155 concerning to the two central/key enzymes investigated, i.e. KstD and Ksh, is less redundant than in *Rhodococcus*. Perhaps, the low redundancy of these central enzymes can explain why many strains currently used at industrial scale to transform phytosterols into AD, ADD or 9OH‐AD are mycobacterial mutants obtained by conventional mutagenesis, i.e. because single mutations like those produced in this work might render a producer strain. Genomic analyses of some of these industrial mutants have confirmed this hypothesis (data not shown).

**Figure 3:**
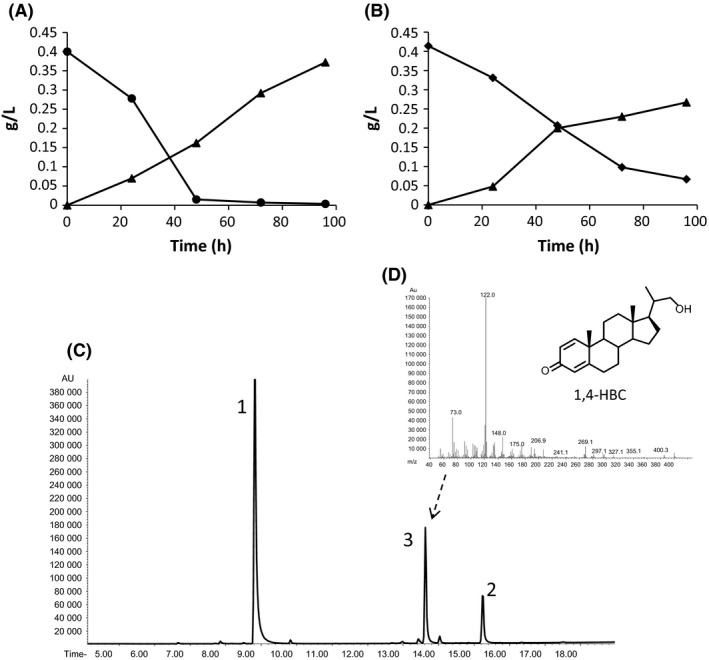
Production of ADD by the MS6039 mutant in shake flasks. ADD is represented by triangles. (A) 9 mM glycerol + 0.4 g l^−1^ of cholesterol (circles) used as substrates; (B) 9 mM glycerol + 0.4 g l^−1^ of phytosterols (diamonds) used as substrates; (C) Analysis by CG‐MS of the products after 96 h of incubation on phytosterols. (1) ADD, (2) cholestenone (internal standard), and (3) 1,4‐HBC. (D) Chemical structure and fragmentation pattern of 1,4‐HBC. The data reported are the averages of three different assays.

**Figure 4:**
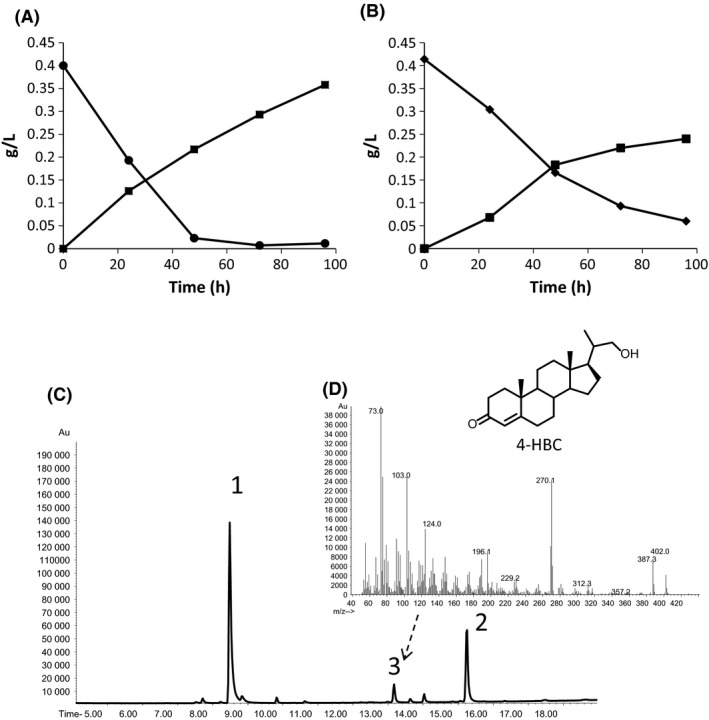
Production of AD by the MS6039‐5941 mutant in shake flasks. AD is represented by squares. (A) 9 mM glycerol + 0.4 g l^−1^ of cholesterol (circles) used as substrates; (B) 9 mM glycerol + 0.4 g l^−1^ of phytosterols (diamonds) used as substrates; (C) Analysis by GC/MS of the products after 96 h of culture on phytosterols. (1) AD, (2) cholestenone (internal standard) and (3) 4‐HBC. (D) Chemical structure and fragmentation pattern of 4‐HBC. The data reported are the averages of three different assays.

Another interesting difference observed between the sterol catabolism of *Mycobacterium* and *Rhodococcus* is the presence of 4‐HBC and 1,4‐HBC alcohols as metabolic by‐products in the cultures of our mutants (Figs 3C, D and 4C, D). It has been described that the cultures of equivalent *Rhodococcus* mutants only accumulate the corresponding acids (Wilbrink *et al*., 2011; Yeh *et al*., 2014). The RG32 mutant of *Rhodococcus rhodochrous* DSM43269, a mutant completely devoid of Ksh by inactivation of five *kshA* genes, was able to produce very small amounts of ADD from β‐sitosterol (7% molar) but large amounts of 3‐oxo‐23,24‐bisnorchola‐1,4‐dien‐22‐oic acid (1,4‐BNC) (67% molar) (Wilbrink *et al*., 2011). A *kshB* ^*‐*^ mutant of *Rhodococcus equi* USA‐18 was also able to produce ADD and 1,4‐BNC from sterols in similar molar ratios (Yeh *et al*., 2014).

In this regard, 4‐HBC alcohol was detected many years ago together with AD during phytosterol transformations performed by the industrial strain of *Mycobacterium* sp. NRRL B‐3805 (Marsheck *et al*., 1972). This alcohol was postulated as a side reaction product but not as a physiological intermediate of the major pathway leading to production of C‐17‐ketonic products (Marsheck *et al*., 1972). It has been proposed that 4‐HBC and 1,4‐HBC derive from 4‐BNC and 1,4‐BNC, respectively, by the action of a carboxyl‐reductase by one or two consecutive enzymatic steps (Szentirmai, 1990; Xu *et al*., 2016). Nevertheless, these carboxyl‐reductases have not been identified in mycobacteria yet (Xu *et al*., 2016). Assuming this hypothesis, these enzymes should not be active, or very low active, in *Rhodococcus*, since only the 4‐BNC/1,4‐BNC acids are detected in this organism (Yeh *et al*., 2014).

The accumulation of these C‐22 intermediates suggests that the elimination of the last isopropyl group of the sterol side‐chain seems to be highly dependent of the 9α‐hydroxylation by Ksh. We assume that in the absence of 9α‐hydroxylation, the enzymes responsible for removing the last propionic acid of the side‐chain at C‐17, i.e. the postulated acyl‐CoA‐dehydrogenase, enoyl‐CoA‐hydratase and aldol‐lyase enzymes (García *et al*., 2012), do not work efficiently. This inefficiency can be caused because they are feedback inhibited by the accumulation of AD and/or ADD, or because the real substrates of these enzymes are the 9α‐hydroxy derivatives of 1,4‐BNC‐CoA or 4‐BNC‐CoA. In this sense, it has been demonstrated that the best substrate of Ksh from *M. tuberculosis* is 4‐BNC‐CoA and 1,4‐BNC‐CoA (Capyk *et al*., 2011), suggesting that 9α‐hydroxylation occurs before the release of the last propionic acid at C‐17. If the previous 9α‐hydroxylation of the steroid is critical to be recognized as a substrate for some of the enzymes involved in the release of the side‐chain, this might explain why in *Rhodococcus* the absence of KshB impairs the complete degradation of the sterol side‐chain (van der Geize *et al*., 2002a). Then, most probably the homologous enzymes of *Mycobacterium* are efficient enough on non‐hydroxylated substrates and thus, the 9α‐hydroxylation of sterols is not so critical to allow the complete side‐chain degradation. In *M. neoaurum* ATCC 25795, the accumulation of 4‐HBC and 1,4‐HBC was only detected after the deletion of the *hsd4A* gene coding for a dual‐functional enzyme with both 17β‐hydroxysteroid dehydrogenase and β‐hydroxyacyl‐CoA dehydrogenase activities. However, these compounds are not the substrates of the Hsd4A *in vitro* (Xu *et al*., 2016). This means that some partial or complete blockage of the side‐chain degradation might cause the accumulation of 4‐HBC or 1,4‐HBC as by‐products. This blockage can be caused not only by specific mutations, but also by retro‐inhibitions of the enzymes due to the accumulation of certain intermediates when the cells are cultured with high concentrations of sterols.

During the course of this work, Xu *et al*. (2015) have described a double (Δ*kshA1*, Δ*kstD1*) mutant of *Mycobacterium* sp. (apparently a derivative of *M. neoaurum* NwIB‐02 (*ΔkstD1*) (Wei *et al*., 2010) that accumulates AD, ADD, 1,4‐HBC and 4‐HBC at different concentrations depending of fermentation temperature when cultured on phytosterols. This result confirmed that mycobacteria accumulate C‐22 alcohols instead of the corresponding C‐22 acids. Interestingly, they have also demonstrated that the residual KstD activity is not active on 9OH‐AD (Xu *et al*., 2015) suggesting that a Δ^1^‐dehydrogenation by KstD precedes Ksh hydroxylation. Remarkably, in contrast with our MS6039‐5941 (*ΔkshB1‐ΔkstD1*) double mutant, this *M. neoaurum* mutant still retains other Ksh and KstD active isoenzymes, because this mutant completely metabolized high amounts of the sterol nucleus (39.8% by moles), which considerably reduces the final conversion yield of the process (Xu *et al*., 2015). This result strongly reinforces our proposal that *M. smegmatis* can be considered as a good cell factory to produce steroid synthons by metabolic engineering.

The finding that MS6039 and MS6039‐5941 mutants can produce large amounts of ADD and AD from natural sterols, respectively, constitutes an experimental demonstration that metabolic engineering can be implemented in a model mycobacterial system like *M. smegmatis* mc^2^155 to generate pharmaceutical steroid synthons at industrial scale using a rational metabolic engineering approach. In fact, these engineered strains are already competing at industrial scale, i.e. under industrial operational conditions, with the existing strains isolated from environmental sources that were modified for their industrial use long time ago by conventional mutagenic procedures. Thus, our results lay the foundations for the valuation of *M. smegmatis* as a useful tool to develop *à la carte* engineered strains as cell factories to transform with high efficiency natural sterols into valuable pharmaceutical steroids.

## Experimental procedures

### Chemicals

Cholest‐4‐en‐3‐one was purchased from Fluka (Steinheim, Germany). ADD and AD were purchased from TCI America. Chloroform and glycerol were purchased from Merck (Darmstardt, Germany). Cholesterol, N,O‐bis(trimethylsilyl) trifluoroacetamide (BSTFA), gentamicin, pyridine, Tween 80 and tyloxapol were from Sigma (Steinheim, Germany). Oligonucleotides were from Sigma‐Genosys.

### Bacterial strains and culture conditions

The strains as well as the plasmids used in this work are listed in Table 2. *M. smegmatis* mc^2^155 and its mutant strains were grown at 37°C in an orbital shaker at 200 r.p.m. Middlebrook 7H9 broth medium (Difco) containing 10% albumin‐dextrose‐catalase supplement (Becton Dickinson, New Jersey, USA) and 0.05% Tween 80 was used as rich medium. 7H10 agar (Difco) plates supplemented with albumin‐dextrose‐catalase were also used for solid media. Middlebrook 7H9 broth medium (Difco) without albumin‐dextrose‐catalase supplements containing 18 mM glycerol was used as a minimal medium. The minimal culture media used for the production of AD or ADD contained a mixture of 9 mM glycerol and 0.4 g l^−1^ cholesterol, or a mixture of 9 mM glycerol and 0.4 g l^−1^ of phytosterols. Commercial phytosterols (provided by Gadea Biopharma, Spain) contained a mixture of different sterols (w/w percentage): brassicasterol (2.16%), stigmasterol (8.7%), campesterol (36.8%) and β‐sitosterol (54.4%). Cholesterol and phytosterols were dissolved in 10% tyloxapol prior to its addition to the minimal medium when assayed in flasks. Due to the low solubility of cholesterol and phytosterols, stock solutions were warmed at 80°C in agitation, sonicated in a bath for 1 h and then autoclaved. Gentamicin (5 μg/ml) was used for selection of *M. smegmatis* mutant strains when appropriate.

**Table 2:** Bacterial strains and plasmids used in this study

Strains or plasmids	Genotype and/or description	Source or reference
Strains		
*Mycobacterium smegmatis*		
mc^2^155	*ept‐1*, mc²6 mutant efficient for electroporation	Snapper *et al*. (1990)
MS6039	*M. smegmatis* mc^2^155 Δ*MSMEG_6039*	This study
MS6039‐5941	*M. smegmatis* mc^2^155 Δ*MSMEG_6039* Δ*MSMEG_5941*	This study
mc^2^155 (pMV261)	mc^2^155 strain harbouring plasmid pMV261	This study
MS6039 (pMV261)	MS6039 strain harbouring plasmid pMV261	This study
MS6039 (pMV6039)	MS6039 strain harbouring plasmid pMV6039	This study
*Escherichia coli*		
DH10B	F^*‐*^, *mcrA*, Δ (*mrrhsdRMS‐mcrBC*), Φ80d*lacZ*ΔM15, Δ*lacX74*,* deoR*,* recA1*,* araD139*, Δ(*ara‐leu*)7697, *galU*,* galK*, λ^‐^, *rpsL*,* endA1*,* nupG*	Invitrogen
Plasmids		
pJQ200x	Suicide vector used to perform allelic exchange mutagenesis in *Mycobacterium*, P15A *ori*,*sacB*, Gm^r^, *xylE*	Jackson *et al*. (2001)
pJQ6039	pJQ200x containing one fragment upstream and another downstream of *MSMEG_6039* gene	This study
pJQ5941	pJQ200x containing one fragment upstream and another downstream of *MSMEG_5941* gene	This study
pGEM^®^‐T Easy	*E. coli* cloning vector; Amp^r^	Promega
pGEMT6039	pGEMT‐Easy harbouring the *MSMEG_6039* gene encoding KshB1 from *M. smegmatis* mc^2^ 155	This study
pMV261	*Mycobacterium*/*E. coli* shuttle vector with the kanamycin resistance *aph* and the promoter from the *hsp6*0 gene from *M. tuberculosis*	Stover *et al*. (1991)
pMV6039	pMV261 harbouring the *MSMEG_6039* gene encoding KshB1 from *M. smegmatis* mc^2^ 155	This study

Bioreactor experiments were performed using the culture media previously described (Herrington and Spassov, 2003) containing a vegetal oil to dissolve phytosterols and corn steep liquor as supplementary carbon and nitrogen sources. The bioconversion experiments were performed in stainless steel (20‐l) or glass (2‐ and 5‐l) jar bioreactors with efficient stirring. The entire process was performed at 37°C.

*Escherichia coli* DH10B strain was used as a host for cloning. It was grown in rich LB medium at 37°C in an orbital shaker at 200 r.p.m. LB agar plates were used for solid media. Gentamicin (10 μg ml^−1^), ampicillin (100 μg ml^−1^) or kanamycin (50 μg ml^−1^), were used for plasmid selection and maintenance in this strain.

### Gene deletions

The knock‐out strains of *M. smegmatis* named MS6039 and MS6039‐5941 were constructed by homologous recombination using the pJQ200x plasmid, a derivative of the suicide pJQ200 vector that does not replicate in *Mycobacterium* (Jackson *et al*., 2001). The strategy consist, for each gene, in generating two fragments of ~700 bp, the first one containing the upstream region and few nucleotides of the 5′end of the gene and the second one containing the downstream region and few nucleotides of the 3′end of the gene, that are amplified by PCR using the oligonucleotides described in Table 3 and *M. smegmatis* genomic DNA as template (Fig. S1). *M. smegmatis* genomic DNA extraction was performed as described (Uhía *et al*., 2011). The two fragments generated were digested with the corresponding enzymes and cloned into the plasmid pJQ200x using *E. coli* DH10B competent cells as described (Uhía *et al*., 2011). Plasmid DNA from *E. coli* DH10B recombinant strains was extracted using the High Pure Plasmid Purification Kit (Roche, Basel, Switzerland), according to the manufacturer's instructions. This procedure was performed for genes *MSMEG_6039* and *MSMEG_5941*, generating the pJQ6039 and pJQ5941 plasmids respectively. Plasmid pJQ6039 was electroporated into competent *M. smegmatis* mc^2^155 to obtain strain MS6039. Plasmid pJQ5941 was electroporated into competent MS6039 cells to obtain MS6039‐5941 strain. Single cross‐overs were selected on 7H10 agar plates containing gentamicin and the presence of the *xylE* gene encoded in pJQ200x was confirmed by spreading catechol over the single colonies of electroporated *M. smegmatis*. The appearance of a yellow coloration indicates the presence of the *xylE* gene. Colonies were also contra‐selected in 10% sucrose. A single colony was grown in 10 ml of 7H9 medium with 5 μg ml^−1^ gentamicin up to an optical density of 0.8–0.9 and 20 μl of a 1:100 dilution was plated onto 7H10 agar plates with 10% sucrose to select for double cross‐overs. Potential double cross‐overs (sucrose‐resistant colonies) were screened for gentamicin sensitivity and the absence of the *xylE* gene. The mutant strains MS6039 and MS6039‐5941 were analysed by PCR and DNA sequencing to confirm the deletions of *MSMEG_6039* and *MSMEG_5941* genes.

**Table 3:** Primers used in this study

Primer	Sequence (5′–3′)^a^	Use
MSMEG_6039 up F	ctagctcgagccagttgtgcacaccgatg	*MSMEG_6039* deletion (amplification of upstream region)
MSMEG_6039 up R	ctagactagtgcagcgtcaggaactggc	*MSMEG_6039* deletion (amplification of upstream region)
MSMEG_6039 down F	ctagactagtggtgcacatggagatcaacg	*MSMEG_6039* deletion (amplification of downstream region)
MSMEG_6039 down R	ctagtctagagtaccagtcgatcggtgtc	*MSMEG_6039* deletion (amplification of downstream region)
MSMEG_5941 up F	ctagactagtgatgttgcgaatgtcgatgtc	*MSMEG_5941* deletion(amplification of upstream region)
MSMEG_5941 up R	ctagtctagaccaccacaacgtcgtactcc	*MSMEG_5941* deletion(amplification of upstream region)
MSMEG_5941 down F	ctagtctagaccatgacattcggttacctgg	*MSMEG_5941* deletion(amplification of downstream region)
MSMEG_5941 down R	ctaggagctcgcagggagatctcgaaatcg	*MSMEG_5941* deletion (amplification of downstream region)

a Restriction sites are underlined.

### Construction of pMV6039 plasmid

To isolate the *kshB1* gene from *M. smegmatis* mc^2^ 155 genomic DNA was extracted and amplified by PCR using the primers MSMEG_6039F (CGGAATTCTGACCTAAGGAGGTGAATGTGACTGATGAGCCCCTGGG) and MSMEG_6039R (CGAAGCTTCTATTCGTCGTAGGTGACTTCG). The amplified fragment of 1092 bp was cloned into the commercial plasmid pGEM^®^‐T Easy to generate pGEMT6039 plasmid that was transformed in *E. coli* DH10B competent cells. The cloned fragment was further digested with *Eco*RI and *Hin*dIII to clone into pMV261, a shuttle plasmid that replicates in *E. coli* and *Mycobacterium* generating the plasmid pMV6039 that was transformed into *E. coli* DH10B to generate the recombinant strain *E. coli* DH10B (pMV6039). Once the sequence of the pMV6039 was checked it was used to transform electrocompetent cells of *M. smegmatis* MS6039 generating the *M. smegmatis* MS6039 (pMV6039) recombinant strain.

### GC/MS analyses

To perform GC/MS analysis, culture aliquots (0.2 ml) were extracted twice at various extents of incubation with an equal volume of chloroform. Previously to its extraction, 10 μl of a solution of 10 mM cholesterol (when phytosterols were used as substrate) or 10 mM cholestenone (when cholesterol was used as substrate) dissolved in chloroform were added to the aliquots as internal standards. The chloroform fraction was concentrated by evaporation and the trimethylsilyl ether derivatives were formed by reaction with 50 μl of BSTFA and 50 μl of pyridine and heating at 60°C for 45 min. Calibration standards were derivatized in the same way. The GC/MS analysis was carried out using an Agilent 7890A gas chromatograph coupled to an Agilent 5975C mass detector (Agilent Technologies, Palo Alto, CA, USA). Mass spectra were recorded in electron impact (EI) mode at 70 eV within the m/z range 50–550. The chromatograph was equipped with a 30 m × 0.25 mm i.d. capillary column (0.25_m film thickness) HP‐5MS (5% diphenyl 95% dimethylpolysiloxane from Agilent Technologies). Working conditions in the sample were as follows: split ratio (20:1), injector temperature, 320°C; column temperature 240°C for 3 min, then heated to 320°C at 5°C min−1.For quantification of the peak area, the quantitative masses were 329 + 458 m/z for cholesterol; 343 + 384 m/z for cholestenone; 382 + 472 m/z for campesterol; 394 + 484 m/z for stigmasterol; 357 + 486 m/z for β‐sitosterol; 244 + 286 m/z for AD and 122 + 284 m/z for ADD in selected ions of monitoring. EI mass spectra and retention data were used to assess the identity of compounds by comparing them with those of standards in the NIST Mass Spectral Database and commercial standards (NIST 2011).

### High‐performance liquid chromatography analyses

To perform high‐performance liquid chromatography (HPLC) analysis, samples (1 g) were withdrawn and extracted with 10 mL of ethyl acetate during two hours in a magnetic stirrer. One aliquot was centrifugated 10 min at 10 000 r.p.m. and supernatant was diluted 1:10 in acetonitrile. Samples were filtered (0.2 μm pore size) prior to the chromatographic analysis. The HPLC analysis was carried out using a Waters liquid chromatograph with a PDA detector system. A Phenomenex C18 column (Nucleosil C18, 100 Å, 250 × 4.6 mm, 5 μm particles) was employed and the temperature of the column was fixed in 50°C. Working conditions were as follows: the mobile phase was a mixture water: acetonitrile: acetic acid (48: 52: 0.1 v/v); flow rate was 1.1 mL min^−1^; the injection volumen was 10 μl. Peaks were monitorized at 240 nm and calibrations were performed using highly purified standards of each compound.

## Conflict of interest

None declared.

## Supporting information


**Fig. S1.** Construction of the mutant strains MS6039 and MS6039‐5941. The polylinker restriction sites of the suicide plasmid pJQ200x are indicated (B, *Bam*HI; Sp, *Spe*I; X, *Xba*I; N, *Not*I; Bs, *Bst*XI; S, *Sac*I).

**Fig. S2.** Production of ADD from phytosterols by the MS6039 mutant in 5‐L jar bioreactor. Analysis by HPLC of the transformation products at 120 h of culture. (1) solvent front; (2) ADD; (3) AD; (4) 1,4‐HBC.

**Fig. S3.** Production of AD from phytosterols by the MS6039‐5941 mutant in 2‐L jar bioreactor. Analysis by GC/MS of the transformation products at 96 h of culture. (1) AD; (2) ADD; (3) 4‐ HBC; (4) 1,4‐HBC; (5) campesterol; (6) stigmasterol; (7) β‐sitosterol.
