# Supplementary figures and images for: Mycobacterium smegmatis is a suitable cell factory for the production of steroidic synthons
Source: Microb Biotechnol. 2016 Nov 2;10(1):138–50. doi: 10.1111/1751-7915.12429 (PMC5270728; doi:10.1111/1751-7915.12429)

## Slide 1
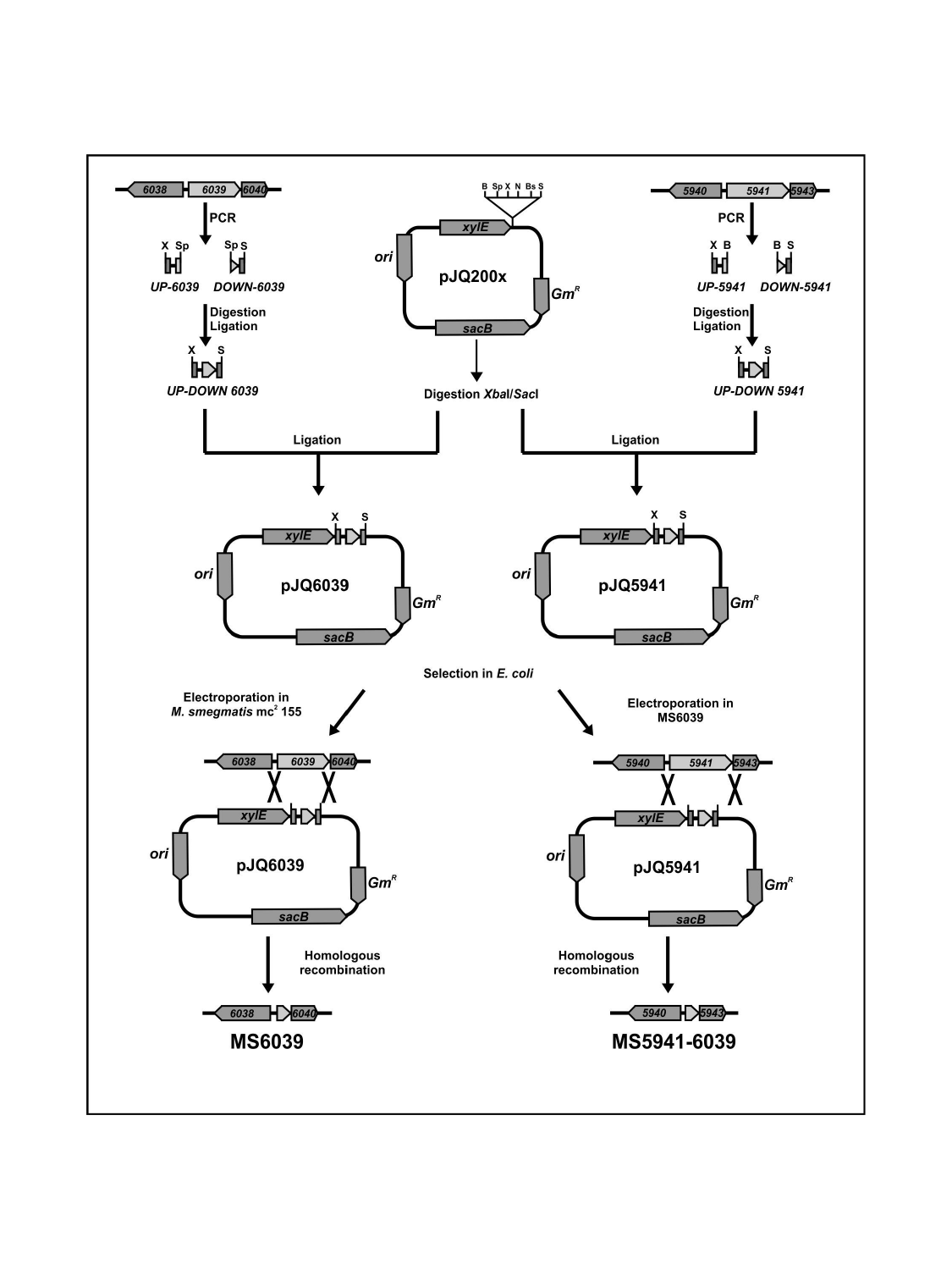

Supplement: Supplementary file 1 — Fig. S1. Construction of the mutant strains MS6039 and MS6039‐5941. The polylinker restriction sites of the suicide plasmid pJQ200x are indicated (B, BamHI; Sp, SpeI; X, XbaI; N, NotI; Bs, BstXI; S, SacI). [file MBT2-10-138-s001.pptx]

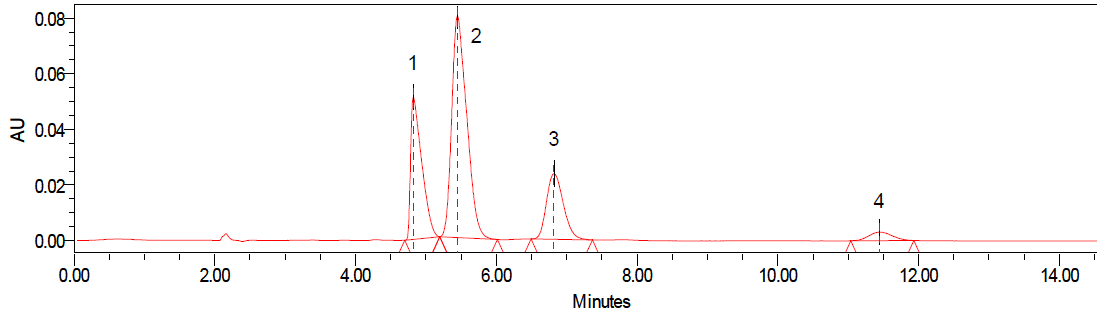


Figure S2

Supplement: Supplementary file 2 — Fig. S2. Production of ADD from phytosterols by the MS6039 mutant in 5‐L jar bioreactor. Analysis by HPLC of the transformation products at 120 h of culture. (1) solvent front; (2) ADD; (3) AD; (4) 1,4‐HBC. [file MBT2-10-138-s002.docx]

## Slide 1
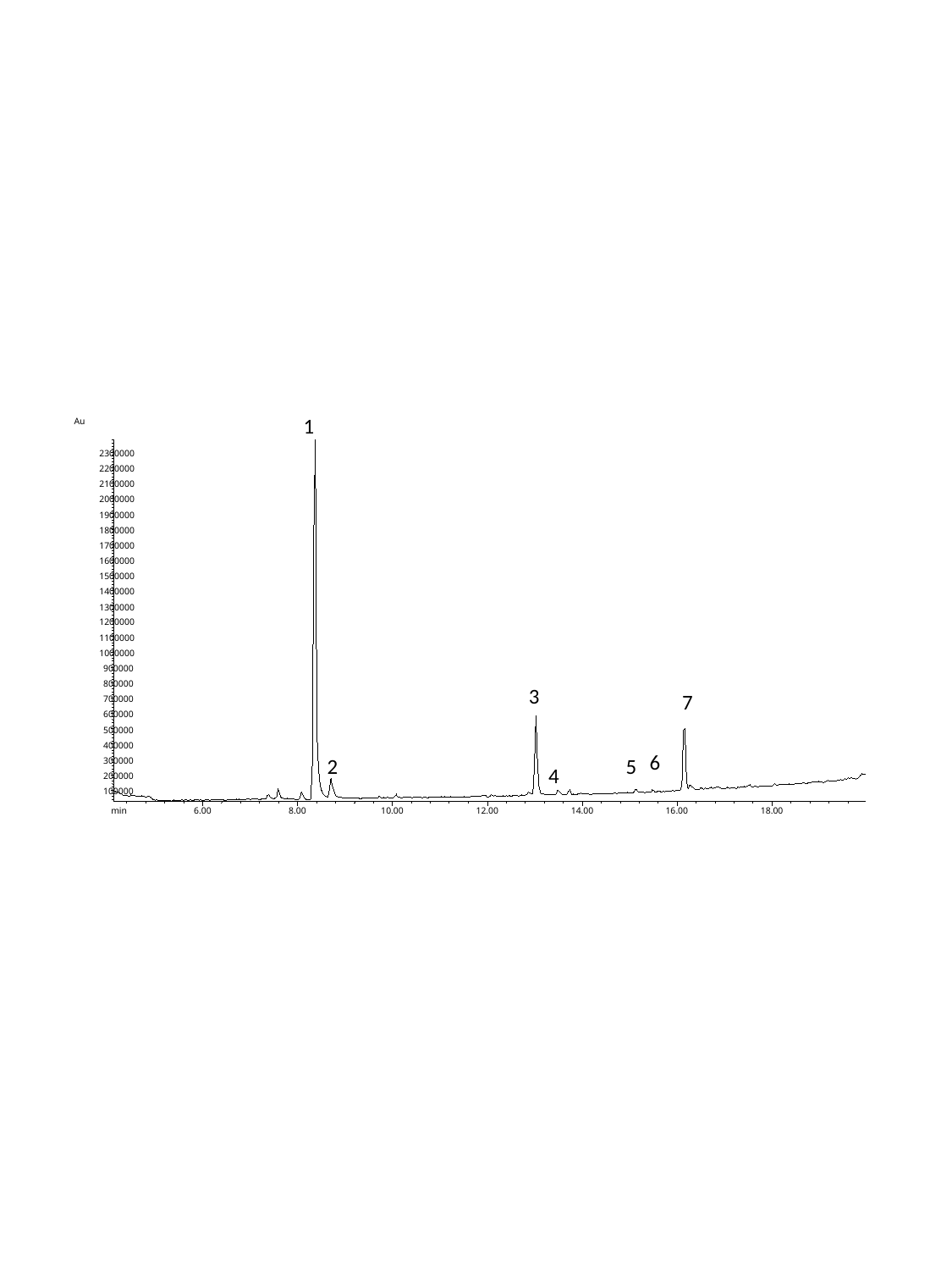

1
Au
2300000
2200000
2100000
2000000
1900000
1800000
1700000
1600000
1500000
1400000
1300000
1200000
1100000
1000000
900000
800000
700000
600000
500000
400000
300000
200000
100000
6.00
8.00
10.00
12.00
14.00
16.00
18.00
min
3
7
6
2
5
4

Supplement: Supplementary file 3 — Fig. S3. Production of AD from phytosterols by the MS6039‐5941 mutant in 2‐L jar bioreactor. Analysis by GC/MS of the transformation products at 96 h of culture. (1) AD; (2) ADD; (3) 4‐ HBC; (4) 1,4‐HBC; (5) campesterol; (6) stigmasterol; (7) β‐sitosterol. [file MBT2-10-138-s003.pptx]
