# Supplementary material for: Mycobacterium smegmatis is a suitable cell factory for the production of steroidic synthons
Source: Microb Biotechnol. 2016 Nov 2;10(1):138–50. doi: 10.1111/1751-7915.12429 (PMC5270728; doi:10.1111/1751-7915.12429)
Supplement: Supplementary file 4 [file MBT2-10-138-s004.docx]

**Supporting information**

Fig. S1. Construction of the mutant strains MS6039 and MS6039-5941. The polylinker restriction sites of the suicide plasmid pJQ200x are indicated (B, *Bam*HI; Sp, *Spe*I; X, *Xba*I; N, *Not*I; Bs, *Bst*XI; S, *Sac*I).

Fig. S2 Production of ADD from phytosterols by the MS6039 mutant in 5-L jar bioreactor. Analysis by HPLC of the transformation products at 120 h of culture. (1) solvent front; (2) ADD; (3) AD; (4) 1,4-HBC.

Fig S3. Production of AD from phytosterols by the MS6039-5941 mutant in 2-L jar bioreactor. Analysis by GC/MS of the transformation products at 96 h of culture. (1) AD; (2) ADD; (3) 4- HBC; (4) 1,4-HBC; (5) campesterol; (6) stigmasterol; (7) β-sitosterol.
